# WNT16B from Ovarian Fibroblasts Induces Differentiation of Regulatory T Cells through β-Catenin Signal in Dendritic Cells

**DOI:** 10.3390/ijms150712928

**Published:** 2014-07-21

**Authors:** Cong-Cong Shen, Yu-Huan Kang, Ming Zhao, Yi He, Dan-Dan Cui, Yu-Yin Fu, Ling-Lin Yang, Lan-Tu Gou

**Affiliations:** 1 State Key Laboratory of Biotherapy/Collaborative Innovation Center for Biotherapy, West China Hospital, Sichuan University, Chengdu 610041, China; E-Mails: shencongcongbio@163.com (C.-C.S.); kangyuhuan@yeah.net (Y.-H.K.); heyidoctor@126.com (Y.H.); fuyuyinbio@yeah.net (Y.-Y.F.); 2 Affiliated Hospital of Luzhou Medical College, Luzhou 646000, China; E-Mail: zhaomingbio@yeah.net; 3 Department of Medical Oncology, the Fifth People’s Hospital of Chengdu, Chengdu 611130, China; E-Mail: cuixinyimd@163.com

**Keywords:** WNT16B, fibroblasts, regulatory T cells, dendritic cells, microenvironment

## Abstract

Treatment for cancer can induce a series of secreted factors into the tumor microenvironment, which can affect cancer progression. Wingless-type MMTV (mouse mammary tumor virus) integration site 16B (WNT16B) is a new member of the WNT family and has been reported to play growth-related roles in previous studies. In this study, we found WNT16B could be expressed and secreted into the microenvironment by human ovarian fibroblasts after DNA damage-associated treatment, including chemotherapy drugs and radiation. We also demonstrated that fibroblast-derived WNT16B could result in accumulation of β-catenin in dendritic cells and secretion of interleukin-10 (IL-10) and transforming growth factor beta (TGF-β), which contributed to the differentiation of regulatory T cells in a co-culture environment. These results shed light on the roles of WNT16B in immune regulation, especially in regard to cancer treatment.

## 1. Introduction

In recent years, much attention has been given to the tumor microenvironment in an effort to better understand and explain the malignant characteristics of cancer [1,2,3]. The tumor microenvironment is an intricate network that is composed of various types of stromal cells such as fibroblasts, lymphocytes, macrophages, dendritic cells, endothelial cells, as well as the extracellular matrix [4,5]. There are dynamic interactions between cancer cells and microenvironment cells, which orchestrate a series of events to promote tumor evolution toward progression, metastasis and immune evasion [6,7,8].

The fibroblast is an important cell type that is known to contribute to tumor progression through the secretion of factors in a paracrine manner [9,10]. These factors such as matrix metalloproteinases (MMPs), hepatocyte growth factor (HGF), and vascular endothelial growth factor (VEGF), play roles in enhancing tumor growth, promoting angiogenesis and generating treatment resistance [2,11,12,13]. More recently, Sun *et al.* [14] found chemotherapy and radiation could induce fibroblasts to secrete a series of factors including wingless-type MMTV (mouse mammary tumor virus) integration site family, member 16B (WNT16B), which was demonstrated to promote the survival of cancer cells and epithelial to mesenchymal transition (EMT) in epithelium through paracrine signalling.

WNT16B is a new member of wingless-related MMTV integration site (WNT) family and has been found to play growth-related roles in previous studies. Binet *et al.* [15] found WNT16B is an overexpressed marker in cells undergoing stress-induced premature senescence. Mazieres *et al.* [16] found that targeted-WNT16B inhibition led to apoptosis of human acute lymphoblastoid leukemia cells using the anti-WNT16B antibody and specific short interfering RNA (siRNA). However, previous studies on WNT16B mainly focused on the field of cell growth, and not its involvement in immune regulation. In this study, we found that WNT16B could be expressed and secreted by ovarian fibroblasts following DNA damage-associated treatment. We further demonstrated that fibroblast-derived WNT16B could activate the canonical β-catenin signal in dendritic cells (DCs) and consequently regulate the regulatory T cells (Tregs) differentiation in a co-culture system. Our data sheds light on WNT16B functions in immune regulation and tumor immune tolerance in addition to its known cell growth functions.

## 2. Results and Discussion

### 2.1. Treatment Induces WNT16B Expression in Ovarian Fibroblast

**Figure 1:**
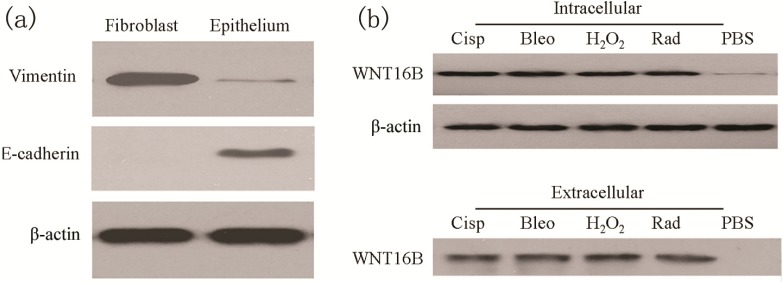
DNA damage-associated treatment to primary ovarian fibroblasts induced expression and secretion of WNT16B. (**a**) Biomarker identification of primary ovarian fibroblasts by Western blot. Fibroblast presented higher level of Vimentin; however, epithelium presented higher level of E-cadherin; (**b**) WNT16B expression and secretion of ovarian fibroblast was induced following DNA damage-associated treatment. Western blot showed that obvious elevation of WNT16B in intracellular space after treatment including cisplatin (Cisp), bleomycin (Bleo), H_2_O_2_ and radiation (Rad). Western blot also showed WNT16B was secreted into the extracellular space after the same treatment (equal volume per well). PBS (phosphate buffered saline) was used as control.

To assess the treatment-induced responses in ovarian fibroblasts, primary ovarian fibroblasts were collected from ovarian tissue and cultured *in vitro*. Vimentin (mesenchymal markers) and E-cadherin (epithelial marker) were examined to distinguish between fibroblasts and epithelial cells, respectively. Western blot showed higher expression of Vimentin in ovarian fibroblasts; however, abundant E-cadherin was detected in ovarian epithelial cells (Figure 1a). After treatment including cisplatin, bleomycin, H_2_O_2_ and radiation, WNT16B of ovarian fibroblasts was obviously detected in cytoplasm and the extracellular space (Figure 1b). These results demonstrated that DNA damage treatment for ovarian fibroblasts could induce WNT16B expression and secretion.

### 2.2. WNT16B from Fibroblasts Activates β-Catenin Pathway in DCs (Dendritic Cells)

**Figure 2:**
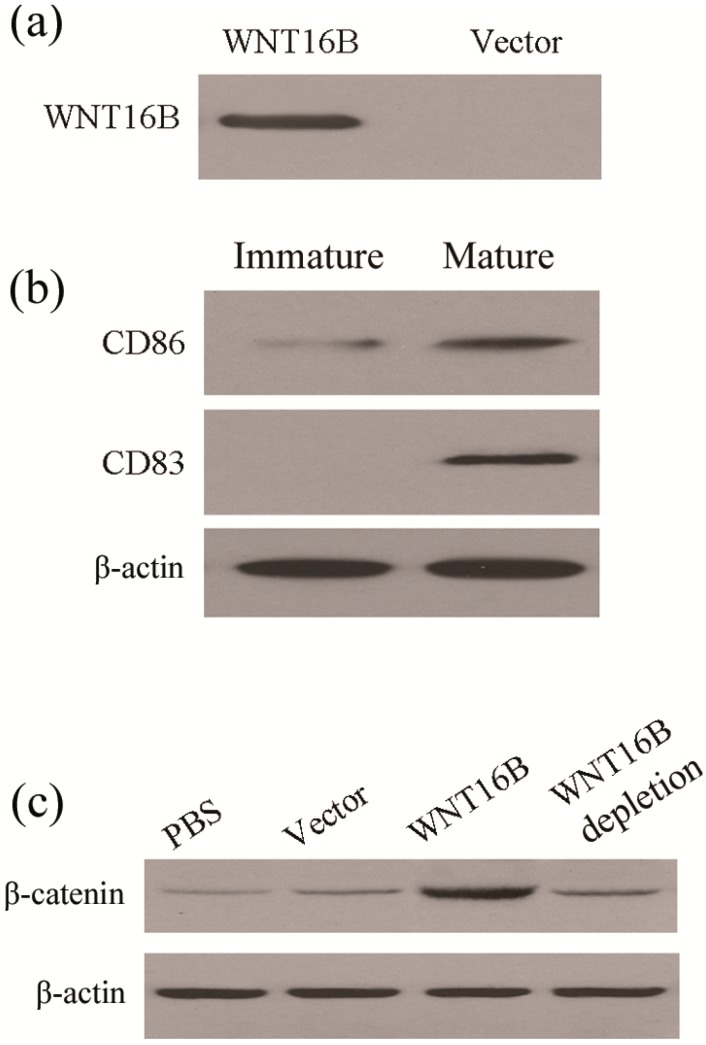
WNT16B from fibroblast activated β-catenin signaling in DCs (Dendritic Cells). (**a**) Western blot showed WNT16B was secreted into culture supernatant by fibroblasts after transfection of recombinant plasmid containing the WNT16B gene; (**b**) Biomarker identification of DCs by Western blot. The levels of CD86 and CD83 were obviously increased in mature DCs; and (**c**) Western blot showed β-catenin was increased in DCs after culture with WNT16B-conditioned medium.

Stabilization of β-catenin is considered to be canonical signal in WNT pathways. On the other hand, activation of β-catenin in DCs has been reported to regulate tolerance. Therefore, we also wanted to know whether WNT16B derived from fibroblasts activate β-catenin in DCs. For this reason, the conditioned medium containing WNT16B was prepared from WNT16B-overexpressing ovarian fibroblasts (Figure 2a). The DCs were separated from peripheral blood mononuclear cells (PBMCs) and cultured as described in the Methods section. These cells were analysed for expression levels of CD86 and CD83 in immature and mature status by Western blot (Figure 2b). When granulocyte-macrophage colony-stimulating factor (GM-CSF) and IL-4 were introduced into the culture medium, the DCs developed into mature status on the fourth day; in the absence of GM-CSF and IL-4 DC development remained at the immature stage.

To explore the effects of WNT16B, the DCs were cultured in X-VIVO serum-free medium supplemented with GM-CSF and IL-4 for 3 days. On the fourth day, the supernatant was carefully removed and replaced with different conditioned medium. After 24 h of culture in different conditioned medium, including phosphate buffered saline (PBS), the levels of vector, WNT16B or WNT16B depletion, β-catenin in DCs were assessed. Western blot assays revealed higher β-catenin levels in DCs cultured with WNT16B-conditioned medium, compared with DCs cultured with PBS and vector-conditioned medium (Figure 2c). However, when WNT16B-depleted medium was used, the β-catenin level in DCs was significantly reduced (Figure 2c). These results indicate that WNT16B from fibroblasts could activate the canonical β-catenin pathway in DCs.

### 2.3. Different Cytokine Secretion of DCs Occurs Following WNT16B Induction

**Figure 3:**
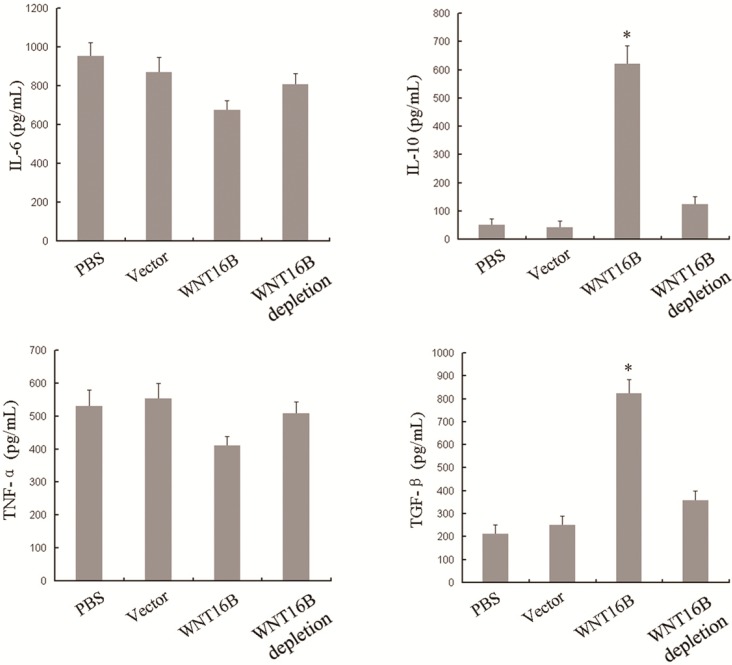
Different cytokine secretion occurred following WNT16B-induced DC differentiation. DCs were cultured in different conditioned medium including PBS, vector, WNT16B or WNT16B depletion, respectively for 3 days. IL-6, IL-10, TNF-α and TGF-β in cell culture supernatant were determined by ELISA (enzyme-linked immunosorbent assay). (Asterisks indicate statistically significant difference *versus* the vector group, *p* < 0.05).

In order to further investigate the potential effects of WNT16B on DCs, we examined the cytokine secretion of DCs after culture in different conditioned medium including PBS, vector, WNT16B or WNT16B depletion, respectively. After 3 days of culture, IL-6, IL-10, TNF-α and TGF-β in cell culture supernatant were determined by enzyme-linked immunosorbent assay (ELISA). Compared with controls (PBS and vector), IL-6 and TNF-α presented no significant changes in culture supernatant from DCs that were cultured in WNT16B-conditioned medium, but IL-10 and TGF-β increased significantly in the culture supernatant from DCs that were cultured in WNT16B-conditioned medium (Figure 3). However, when WNT16B-depleted medium was used, IL-10 and TGF-β levels nearly recovered to control levels (Figure 3). These results indicated WNT16B could induce the different cytokine-secretion of DCs, and the induction occurred in a WNT16B-dependent manner.

### 2.4. Tregs Differentiation

**Figure 4:**
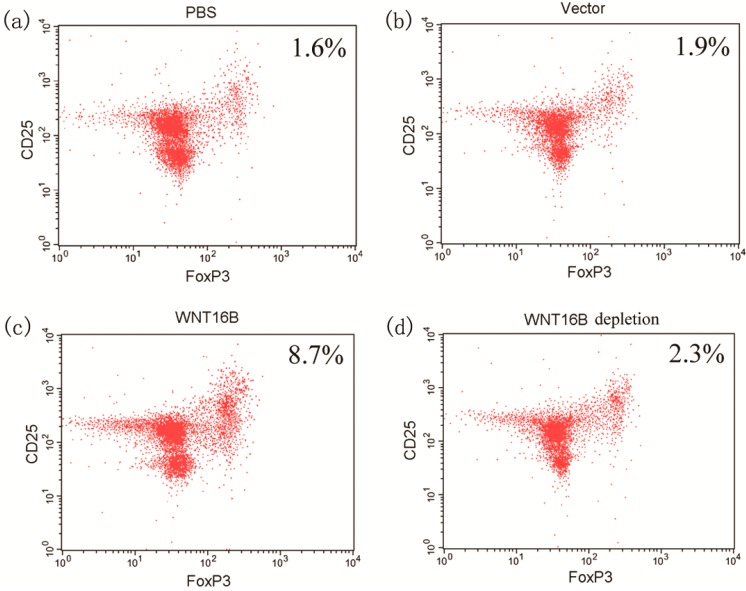
WNT16B induced Tregs differentiation through DCs. After co-culture of PBMCs and DCs, flow cytometry was used to assess the percentage of CD4+CD25+Foxp3+ T cells. Compared with PBS (**a**) and vector-conditioned medium (**b**); the percentage of Tregs under WNT16B-conditioned co-culture was significantly increased (**c**); However, when WNT16B-depleted medium was used, the percentage of Tregs significantly recovered (**d**).

Since the characteristic cytokine-secretion of DCs occurred after culture with WNT16B-conditioned medium, we further assessed the potential effects on Treg differentiation by DCs under the conditions of WNT16B existence. After 5 days of co-culture of PBMCs and DCs and the subsequent 3 days of expansion in the presence of CD3 antibody, CD28 antibody and IL-2, flow cytometry was used to assess the percentage of CD4+CD25+Foxp3+ T cells. Compared with PBS (1.6%) and vector-conditioned medium (1.9%), the percentage of Tregs under WNT16B-conditioned co-culture was significantly increased (Figure 4), up to 8.7%. However, when WNT16B-depleted medium was used, the percentage of Tregs significantly decreased to 2.3% (Figure 4). Above data demonstrated that WNT16B derived from fibroblasts could regulate Tregs differentiation through DCs, and the Tregs differentiation was also inhibited by WNT16B depletion.

### 2.5. Discussion

In this study, we focused on the potential effects of WNT16B in the cellular network of tumor microenvironment using a co-culture system of fibroblasts and DCs. We demonstrated DNA damage-associated treatment could induce WNT16B expression and secretion of ovarian fibroblasts. We further assessed the characteristic cytokine secretion of DCs and provided evidence concerning DC-induced Tregs in the presence of WNT16B and revealed a new aspect of WNT16B function in the area of immune regulation; interestingly, this function may play a role in tumor progression through immune evasion.

Fibroblasts are an essential cell type in the tumor microenvironment, which could produce a number of important cytokines to support the growth of tumor cells [17,18]. Straussman *et al.* [2] found that fibroblasts could secrete HGF in tumor microenvironment to elicit resistance of tumor cells to treatment of rapidly accelerated fibrosarcoma (RAF) inhibitors through HGF/mesenchymal-epithelial transition factor (MET) and its downstream signaling pathways such as mitogen activated protein kinase (MAPK) and phosphoinositide 3-kinase/protein kinase B (PI(3)K/AKT) signaling. Subsequently, dual inhibition of RAF and HGF/MET resulted in reversal of drug resistance, suggesting that combined therapy of anticancer drugs plus inhibitors of microenvironment factors is a potential therapeutic strategy for cancer. On the other hand, since fibroblasts are adjacent to tumor cells in the microenvironment, anti-tumor treatment usually affects peripheral cells including fibroblasts [14,19,20]. Yu Sun *et al.* [14] found that fibroblasts could produce WNT16B following DNA-damage treatment such as H_2_O_2_, bleomycin and gamma radiation, which activated the canonical WNT/β-catenin pathway in prostate cancer cells, eventually resulting in cancer growth and therapy resistance. 

An important function of fibroblasts is the repair of tissue injury [21,22,23]. In some cases of repair, disordered secretion of cytokines from fibroblasts occurs, often causing side effects. For radiotherapy of cancer, the radiation usually damages fibroblasts, inducing increased release of several factors such as TGF-β and PDGF, and resulting in abnormal accumulation of ECM and finally the formation of pulmonary fibrosis [24,25,26]. In this study, we found that ovarian fibroblasts could produce and secrete WNT16B into the microenvironment after DNA damage-treatment. It is noteworthy that the low dose of treatment was introduced in our experiments, which could not completely kill fibroblasts, but switch on its repair process. Unlike previous perspectives concerning the proliferation to cancer cells, we found fibroblast-derived WNT16B could regulate DCs, another important cell type in the tumor microenvironment, through the activation of the canonical Wnt/β-catenin pathway in DCs. Our findings demonstrate a cross-talk between fibroblasts and DCs through factors in the microenvironment following anticancer treatment, which suggests that WNT16B could be involved in immune regulation in addition to its growth-promoting effect on tumor cells.

Activation of β-catenin in DCs was reported to regulate immune tolerance in previous studies [27,28]. Manicassamy *et al.* [29] found that β-catenin activation in DCs was required for the production of anti-inflammatory factors such as retinoic acid-metabolizing enzymes, IL-10 and TGF-β that stimulate Tregs to suppress immunity. Since β-catenin activation mediated by WNT16B was also found in DCs in our study, we further focused on the secretion of various cytokines including IL-6, IL-10, TNF-α and TGF-β from DCs. The characteristic changes of IL-10 and TGF-β prompted us to further explore Tregs induction in co-culture system, because these cytokines are essential to Tregs differentiation [30,31,32]. As we speculated, Tregs induction coincided with characteristic cytokine profiling of DCs under conditioned medium containing WNT16B from fibroblasts. Our data suggested that treatment-induced WNT16B from ovarian fibroblasts could suppress immunity through Tregs that were induced by a series of cytokines from DCs in the tumor microenvironment. Our findings shed light on a new function of WNT16B involved in immune regulation in the tumor microenvironment, which provides a reference on anticancer strategy. In addition to cancer cells, treatment-induced WNT16B from fibroblasts in the tumor microenvironment should also be taken into account.

In summary, we found WNT16B expression and secretion of ovarian fibroblasts could be induced following DNA damage-associated treatment. We demonstrated that a signaling pathway involving WNT16B/β-catenin in DCs could regulate Tregs differentiation in the microenvironment. The present study suggested that WNT16B could be critical for maintaining DCs in a tolerogenic state through induction of various anti-inflammatory factors such as IL-10 and TGF-β. Our data also indicated the suppression of WNT16B could be an attractive strategy for improvement in tumor immunity, especially in the course of treatment associated with DNA damage.

## 3. Experimental Section

### 3.1. Materials

Cisplatin, Bleomycin (BLEO) and hydrogen peroxide (H_2_O_2_) were from Sigma-Aldrich (St. Louis, MO, USA). The open reading frames of human WNT16B (GenBank entry: NM_057168.1) were subcloned into pcDNA3.1(+) (Invitrogen, Carlsbad, CA, USA) between BamHI and XhoI sites to obtain recombinant plasmid pc3.1-WNT16B. Primary antibodies were from Abcam (Cambridge, MA, USA) (Vimentin, E-cadherin, CD83 and CD86), Santa Cruz (Dallas, TX, USA) (WNT16B and β-catenin) and eBioscience (San Diego, CA, USA) (CD3 and CD28), respectively. Horseradish peroxidase-conjugated secondary antibodies against immunoglobulin of mouse or rabbit were from Santa Cruz. Human granulocyte-macrophage colony-stimulating factor (GM-CSF), interleukin-4 (IL-4) and interleukin-2 (IL-2) were from Gibco (Carlsbad, CA, USA). ELISA sets to assess interleukin-6 (IL-6), interleukin-10 (IL-10), tumor necrosis factor-α (TNF-α) and transforming growth factor-β (TGF-β) were from R&D Systems (Minneapolis, MN, USA).

### 3.2. Isolation of Ovarian Fibroblasts

Fresh tissues of ovarian cancer were obtained from nine ovarian cancer patients who underwent surgical resection, and the adjacent normal ovarian tissues were carefully separated under sterile conditions. After removal of extraneous tissues, the ovarian tissues were minced to 1-mm pieces and washed in sterile PBS for several times until the supernatant was clear. Then, the pieces were suspended in sterile PBS containing 0.5% trypsin, and incubated with gentle stirring at 37 °C for 20 min. The digestion was terminated by supplement of the equal volume of Dulbecco’s modified eagle’s medium (DMEM) containing 10% fetal calf serum. Dispersed cells were collected by filtration through a sterile stainless steel 150 µm-strainer. The collected cells were washed twice with DMEM containing 10% fetal calf serum and re-suspended in this media for culture in a 5% CO_2_ incubator at 37 °C. After 2 h, unattached epithelial cells were removed to another plate by washing with fresh media, and attached cells were cultured for 24 h. The above steps were repeated twice to fully remove unattached cells, and the remaining cells were continued to be cultured until passage. After four passages, the ovarian fibroblasts were examined and used in the further experiments. The study was approved by the Institutional Ethics Committee of Sichuan University.

### 3.3. Cell Treatment

Ovarian fibroblasts were seeded in a 10-cm dish at a density of 5 × 10^4^ cells/cm^2^. The cells were grown until 80% confluent and respectively treated with 5 µM cisplatin (Cisp), 5 µM Bleomycin (BLEO), 10 µM hydrogen peroxide (H_2_O_2_) for 2 h, or a single radiation dose of 2 Gy with X-rays. After treatment, the cells were rinsed 3 times with DMEM containing 10% fetal calf serum and left to recover for 3 days in medium. Finally, the culture supernatant was subjected to Western-blot to analyze WNT16B expression and secretion. The experiments were performed in triplicate.

### 3.4. Preparation of Conditioned Medium

Ovarian fibroblasts were plated on a 10-cm dish at a density of 5 × 10^4^ cells/cm^2^, and cultured in X-VIVO serum-free medium (Lonza, Basel, Switzerland). The cells were grown to 90%–95% confluence, and transiently transfected with pc3.1-WNT16B using Lipofectamine 2000 (Invitrogen) according to the instructions of manufacturer. PBS and vector were used as controls to assess the effects of WNT16B. At 72 h after transfection, cell culture supernatants were collected, half of which was used to flow through a spin column-coupled with WNT16B antibody in order to deplete WNT16B in culture supernatants. The above two kinds of culture supernatants were mixed with an equal volume of X-VIVO medium, which was used as conditioned medium and stored at 4 °C for next applications.

### 3.5. DCs Culture and Cytokine Assay

Peripheral blood mononuclear cells (PBMCs) were isolated from the blood of the same ovarian cancer patients by the lymphocyte separation medium (Haoyang Ltd., Beijing, China). Briefly, blood samples containing heparin were centrifuged (450× *g*, 10 min, 4 °C), and the cells in middle layer were collected and mixed with an equal volume of lymphocyte separation medium. After another centrifugation (450× *g*, 30 min, 4 °C), the cells in middle layer were collected and washed twice with X-VIVO medium. Then, the PBMCs (2 × 10^5^ cells/well) were plated in a 6-well plate and cultured in X-VIVO medium supplemented with 50 ng/mL GM-CSF and 50 ng/mL IL-4. After 24 h of culture, the supernatant was carefully removed and replaced with X-VIVO medium supplemented with the same GM-CSF and IL-4. On the fourth day, the supernatant was carefully removed and replaced with X-VIVO medium or different conditioned medium from transfected fibroblasts including PBS, vector, WNT16B-conditioned medium or WNT16B-depleted medium. These types of medium were supplemented with the same factors as described above. On the ninth day, the cells were respectively collected and subjected to Western blot and co-culture with PBMCs to induce Tregs. At the same time, the collected supernatants were subjected to analysis of IL-6, IL-10, TNF-α and TGF-β through ELISA according to the manufacturer’s instructions. Three independent experiments were repeated to assess the factors.

### 3.6. Western Blot

The cells or supernatant were collected, lysed in radio-immunoprecipitation assay (RIPA) buffer on ice, and centrifuged at 12,000× *g* for 30 min at 4 °C to extract the proteins in supernatant. The extracted proteins were electrophoresed by 12% sodium dodecyl sulfate-polyacrylamide gel electrophoresis (SDS-PAGE) under denaturing conditions, and subsequently transferred onto PVDF (polyvinylidene difluoride) membranes (Bio-Rad, Hercules, CA, USA). The membranes were blocked in phosphate buffered saline with Tween 20 (PBST) containing 5% skimmed milk for 2 h, washed with PBST three times, and incubated with primary antibodies for 2 h at room temperature. Then, the membranes were washed with PBST three times and incubated with secondary antibodies for 1 h at room temperature. After washing the membranes three times with PBST, the immunoblots were visualized with Immobilon Western Chemiluminescent reagent (Millipore, Billerica, MA, USA). Three independent experiments were repeated to assess the relative protein abundance.

### 3.7. Co-Culture of PBMCs (Peripheral Blood Mononuclear Cells) and DCs

The preparation of PBMSc and DCs were described in the previous steps. The PBMCs were co-cultured with DCs that prepared as described for six days at a ratio of 1:1 in X-VIVO medium or different conditioned medium from treated fibroblasts including PBS, vector, WNT16B-conditioned medium or WNT16B-depleted medium. Then, the cells were given 1 µg/mL anti-CD3, 1 µg/mL anti-CD28 and 500 U/mL IL-2, and cultured for 3 days. For flow cytometry, the cells were labeled with CD4-FITC, CD25-APC and Foxp3-PE (eBioscience) according to the manufacturer’s instructions. CD4+CD25+Foxp3+ Tregs were analysed using a FACScalibur (BD Biosciences, Franklin Lakes, NJ, USA) with CELLQUEST software (BD Biosciences). Three independent experiments were repeated to assess the CD4+CD25+Foxp3+ Tregs.

## 4. Conclusions

WNT16B secretion from ovarian fibroblasts can be induced by DNA damage-associated treatment. WNT16B triggers canonical β-catenin signalling in DCs and induces characteristic cytokine secretion of DCs, which can regulate Tregs differentiation.
